# A comparative study on dispersed doses during photon and proton radiation therapy in pediatric applications

**DOI:** 10.1371/journal.pone.0248300

**Published:** 2021-03-10

**Authors:** Mehrdad Shahmohammadi Beni, Dragana Krstic, Dragoslav Nikezic, Kwan Ngok Yu

**Affiliations:** 1 Department of Physics, City University of Hong Kong, Kowloon Tong, Hong Kong; 2 Faculty of Science, University of Kragujevac, Kragujevac, Serbia; University of Nebraska Medical Center, UNITED STATES

## Abstract

The Monte Carlo method was employed to simulate realistic treatment situations for photon and proton radiation therapy for a set of Oak Ridge National Laboratory (ORNL) pediatric phantoms for 15, 10, 5 and 1-year olds as well as newborns. Complete radiotherapy situations were simulated using the previously developed NRUrad input code for Monte Carlo N-Particle (MCNP) code package. Each pediatric phantom was irradiated at five different positions, namely, the testes, colon, liver, left lung and brain, and the doses in targeted organs (*D*_*t*_) were determined using the track length estimate of energy. The dispersed photon and proton doses in non-targeted organs (*D*_*d*_), namely, the skeleton, skin, brain, spine, left and right lungs were computed. The conversion coefficients (*F = D*_*d*_*/D*_*t*_) of the dispersed doses were used to study the dose dispersion in different non-targeted organs for phantoms for 15, 10, 5 and 1-year olds as well as newborns. In general, the *F* values were larger for younger patients. The *F* values for non-targeted organs for phantoms for 1-year olds and newborns were significantly larger compared to those for other phantoms. The dispersed doses from proton radiation therapy were also found to be significantly lower than those from conventional photon radiation therapy. For example, the largest *F* values for the brain were 65.6% and 0.206% of the dose delivered to the left lung (*P*_*4*_) for newborns during photon and proton radiation therapy, respectively. The present results demonstrated that dispersion of photons and generated electrons significantly affected the absorbed doses in non-targeted organs during pediatric photon therapy, and illustrated that proton therapy could in general bring benefits for treatment of pediatric cancer patients.

## Introduction

In the United States, 3,000 out of 12,000 pediatric cancer patients each year required radiation therapy as their main treatment scheme [1]. The dose delivered to non-targeted normal organs or tissues is referred to as the “dispersed dose”. Considering the large number of pediatric cancer patients each year alone in the United States, it is vital to address the interest of patient-specific and even organ-specific dosimetry in pediatric applications [2]. The risk for pediatric applications was particularly relevant due to their increased radio-sensitivity [3–8]. Specifically, pediatric patients are more vulnerable to radiation-induced risks than adult patients mainly due to their swiftly growing tissues and increased cellular distribution of skeletal active marrow [2]. In this connection, Vassileva *et al*. [9] conducted studies on large pediatric population and noted that the increase in the cancer incidence could be due to ionizing radiations from computed tomography, although the radiation doses were relatively small compared to those used during the radiation therapy. This demonstrated the importance of dispersed photon doses to non-targeted organs in pediatric radiotherapy, where the doses were significantly higher and therefore led to a higher cancer incidence. The risk of secondary cancer induction and subsequent radiation damages could also be evaluated when one had the precise quantitative knowledge of dispersed doses in non-targeted organs. Precise determination of the absorbed dose during radiotherapy to targeted and non-targeted organs would be important, considering the potential induction of secondary cancers by unintended radiation doses [10–12].

The two widely used pediatric radiotherapy modalities are based on the use of photons or protons as the primary particles to deliver the desired amount of dose to the targeted volume. The conventional radiation therapy modality focuses on the use of photons. The concept of using high-energy protons as treatment mode of cancer patients was first proposed by Dr. Robert Wilson in 1946 [13]. Proton therapy exploits the Bragg peak that leads to small lateral dispersion of protons in the targeted tissues. According to Sengbusch *et al*. [14], operating proton facilities were large and had high cost (~$100 million), where a cyclotron or synchrotron was used for particle acceleration. Due to the high cost of these facilities, proton therapy facilities are less abundant when compared to conventional photon ones. However, the crucial question would be whether such large and expensive facilities are advantageous in terms of dose dispersion in pediatric radiotherapy when compared to conventional photon ones.

The highest percentage of pediatric solid tumors are those formed in the central nervous system, and radiation therapy was found to contribute to most of the undesirable late effects such as the effect on the intelligence quotient for pediatric patients having undergone cranial radiation therapy [15]. Clinical studies have demonstrated that the proton therapy technique could limit long-term side effects in pediatric intracranial tumors [16, 17]. The decrease in neurocognitive function in childhood cancer survivors as a function of dose volume and intensity and the age were also previously reported [18].

In conventional photon radiotherapy, unintended radiation doses are unavoidable, which can be scattered from different parts of the treatment room such as floor, walls and the ceiling or from the head section (i.e., positions at which collimators are located) of the linear accelerator, or even from the patient’s own body [19–21]. Notably, experimental determination of doses dispersed to the patient’s non-targeted organs represents a challenging task. In our previous work, the concept of conversion coefficients for determining the dispersed photon doses during radiotherapy was introduced [22], where the doses dispersed to non-targeted organs in an adult male human phantom adopted from the Oak Ridge National Laboratory (ORNL) were characterized [23]. The conversion coefficient was defined by *F = (D*_*d*_*/D*_*t*_*)*, where *D*_*d*_ was the dose dispersed to the non-targeted tissue and *D*_*t*_ was the absorbed dose in the targeted tissue. In the present work, we determined the conversion coefficients for the series of five ORNL pediatric phantoms, namely, newborn, 1, 5, 10 and 15-years old phantoms [24, 25] for modelling photon and proton therapy conditions, which provided quantitative results for assessing the dispersed photon and proton doses in young patients. The conversion coefficients obtained in the present work enabled better treatment planning of young cancer patients, especially in the choice of radiation therapy mode (i.e., photon or proton irradiation). In relation, a quantitative comparison between the effectiveness of photon and proton radiation therapy in terms of particle transport and dispersion in pediatric patients could be made.

Various radiation treatment fields were simulated using the Monte Carlo (MC) technique for a variety of models of medical linear accelerators for photon radiation therapy such as Varian, Siemens and Elekta [26, 27]. In the current study, our previously developed and benchmarked NRUrad input code [22] for Monte Carlo N-Particle (MCNP) code packages was used to simulate realistic radiotherapy scenarios for conventional photon radiation therapy. The readers are referred to ref. [22] and references therein for the complete simulation setup and more details regarding the use of MC method in photon radiation therapy. For the proton therapy mode, irradiation of the set of ORNL pediatric phantoms by the proton beam at National Cancer Center Hospital East (NCCHE), Kashiwa, Chiba, Japan was simulated [28].

In the study by Verhey *et*. *al*. [29] which compared different modalities, it was revealed that protons delivered less dosage to the brain (one of the critical organs) as compared to gamma-knife and linac machines. In another study [30] which discussed different treatment modalities to minimize radiation dispersion to critical organs, it was found that image-guided and three-dimensional conformal radiation therapies could be employed to track daily changes in the size and shape of the tumor and to deliver the radiation dose as precisely as possible to the targeted organ. The intensity modulated radiation therapy (known as IMRT) would move and modulate the beam intensity to deliver varying doses to different regions of the tumor, taking into account its shape and size. In addition, proton therapy was found to be advantageous in delivering most of the radiation dose to the target, while minimizing the damage to the surrounding healthy tissues.

In the present work, the dose distributions among various important tissues in pediatric patients, including the skeleton, skin, brain, spine, left and right lungs were studied. The present input codes and results are useful for planning pediatric radiotherapy, for subsequent risk assessment due to increased sensitivity and for assessing potential radiation damages inflicted on this particular group of patients upon the employment of photon and proton therapy.

## Materials and methods

### Modelled pediatric phantoms

**Fig 1:**
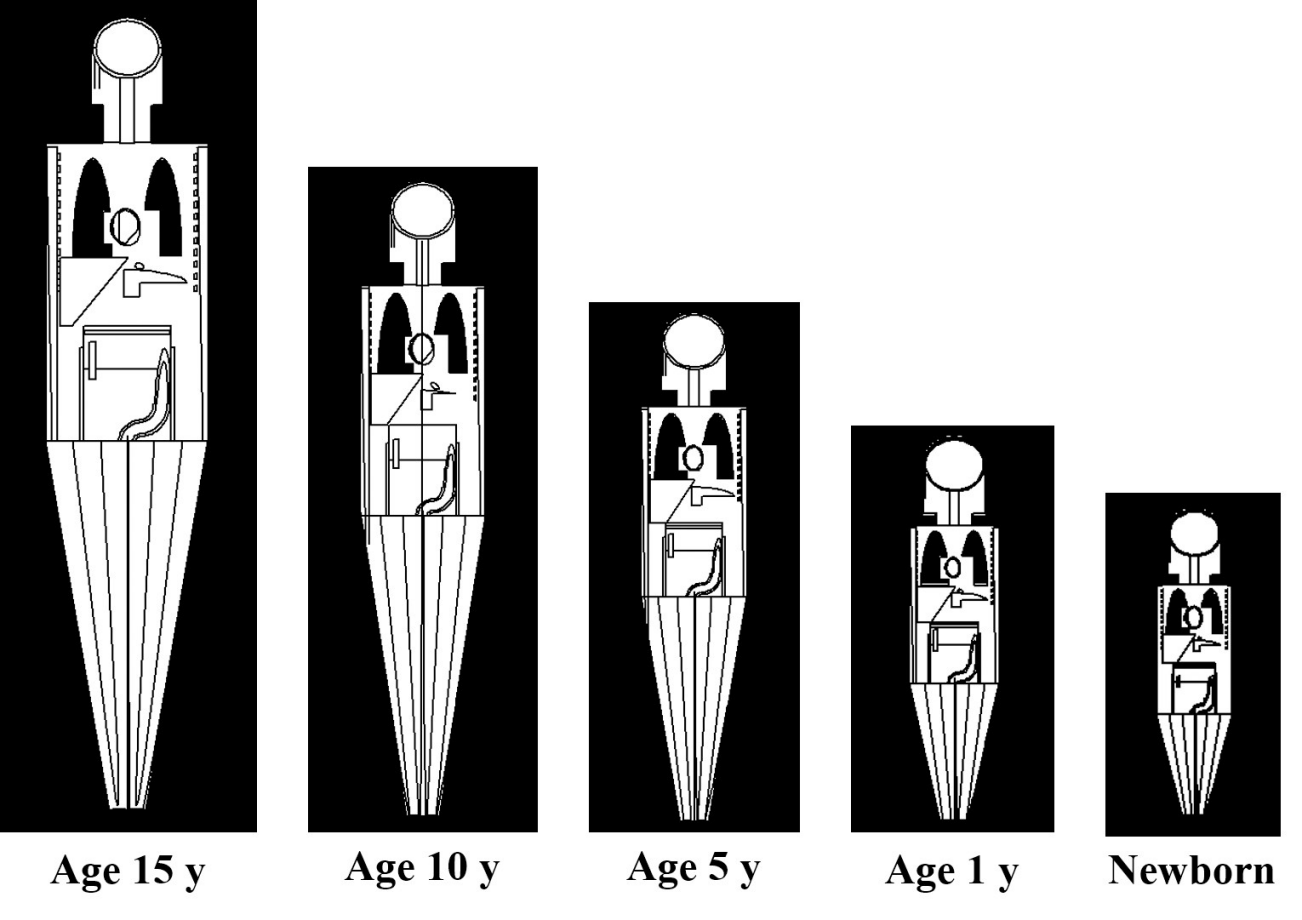
Longitudinal cross-section of ORNL phantoms [23] perpendicular to the incident particle beam.

**Fig 2:**
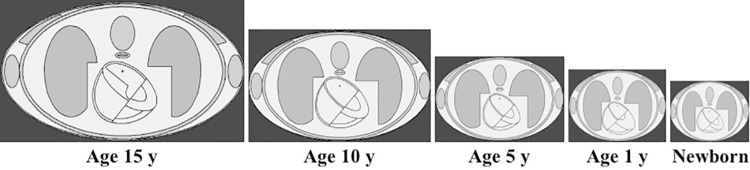
Lateral cross-section of ORNL phantoms [23] perpendicular to the linear accelerator beam.

The pediatric phantoms employed in the present work were those for newborns and 1, 5, 10 and 15-years olds, which were adopted from ref. [23]. These phantoms are schematically shown in Fig 1. Similar to our previous work, five different target organs, namely, testes (*P*_*1*_), colon (*P*_*2*_), liver (*P*_*3*_), left lung (*P*_*4*_) and brain (*P*_*5*_) were chosen for each pediatric phantom. The schematic diagram shown in Figs 1 and 2 represents the longitudinal and lateral cross-section of the modelled phantoms.

The present computations were performed in parallel on a computer cluster using the MPI (Message Passing Interface) build of MCNP [31]. The employed computer cluster consisted of 22 nodes, each equipped with Intel^®^ Xeon^®^ E5-2670 2.60 GHz and interconnected using the InfiniBand QDR (Quad Data Rate). The MPI build of MCNP was built with GNU FORTRAN compiler version 4.4 on CentOS (Community Enterprise Operating System) version 6.8 Linux distribution. The MPICH2 package was used for execution of the code in parallel [32]. Due to the large number of details required for simulating the realistic situation of radiotherapy in the NRUrad input code, parallel computing was needed if the computations should be completed within a feasible time.

### Photon beam therapy using linear accelerator

**Fig 3:**
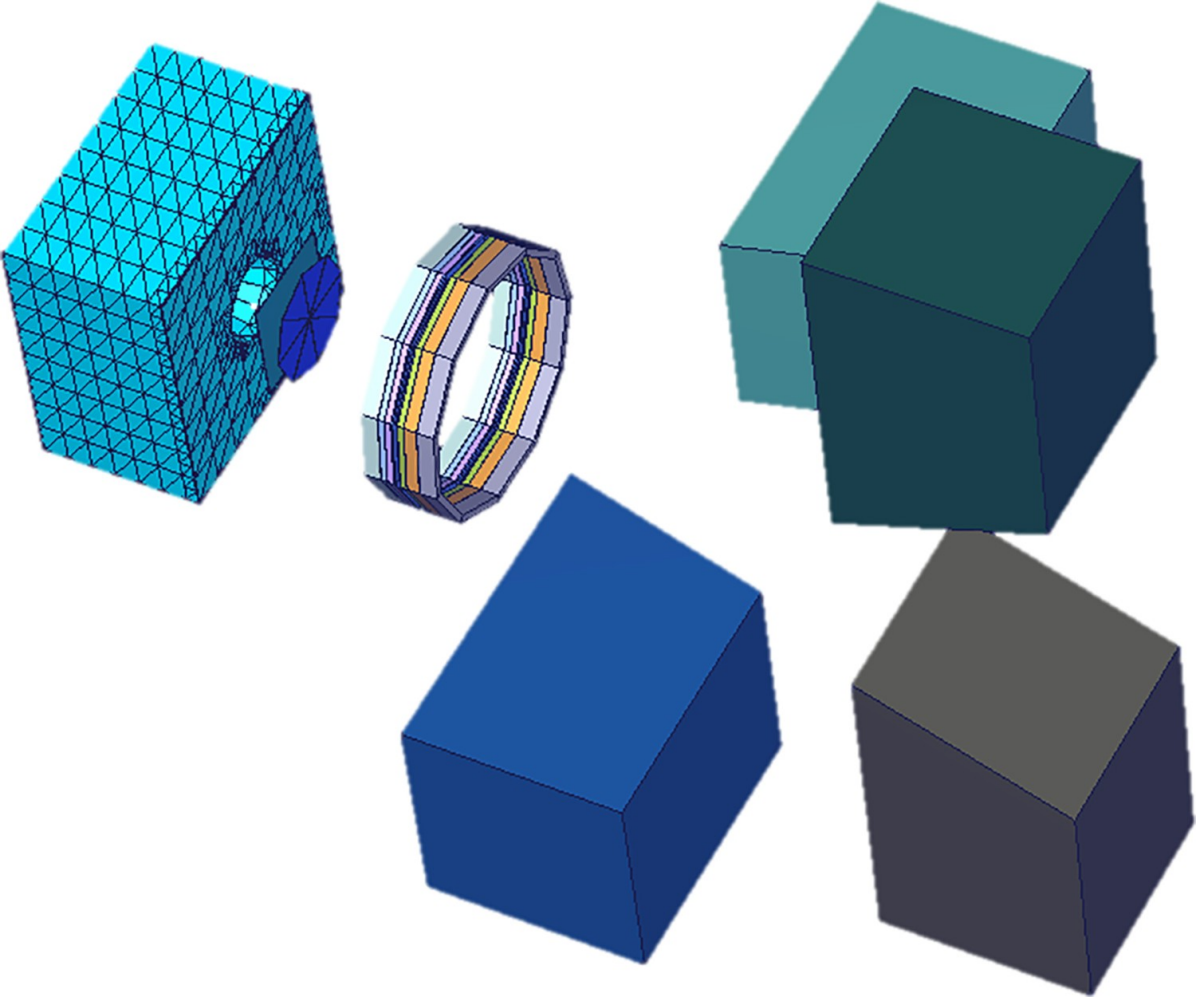
Modelled Varian Clinac 2300 C/D linear accelerator head.

In our previous work, a complete model of photon radiation therapy was modelled with the presence of linear accelerator (linac), radiotherapy room, treatment couch and human phantom [22]. A similar model was used to irradiate the set of pediatric phantoms in the present work as shown in Fig 1. The track length estimate of energy tally was used to calculate the doses delivered to the targeted and non-targeted organs from a 6 MV linac beam. The ENDF/B.VI release 8 photo-atomic data were used in the present computations. Furthermore, the dose values obtained from the present MC model were normalized by the number of primary particles (in this case photons), so explicit evaluation of the particle fluence (*Φ*) was not required (see refs. [33, 34]). The Varian Clinac 2300 C/D linear accelerator head was modelled and shown schematically in Fig 3.

### Proton beam therapy using NCCHE beamline

**Fig 4:**
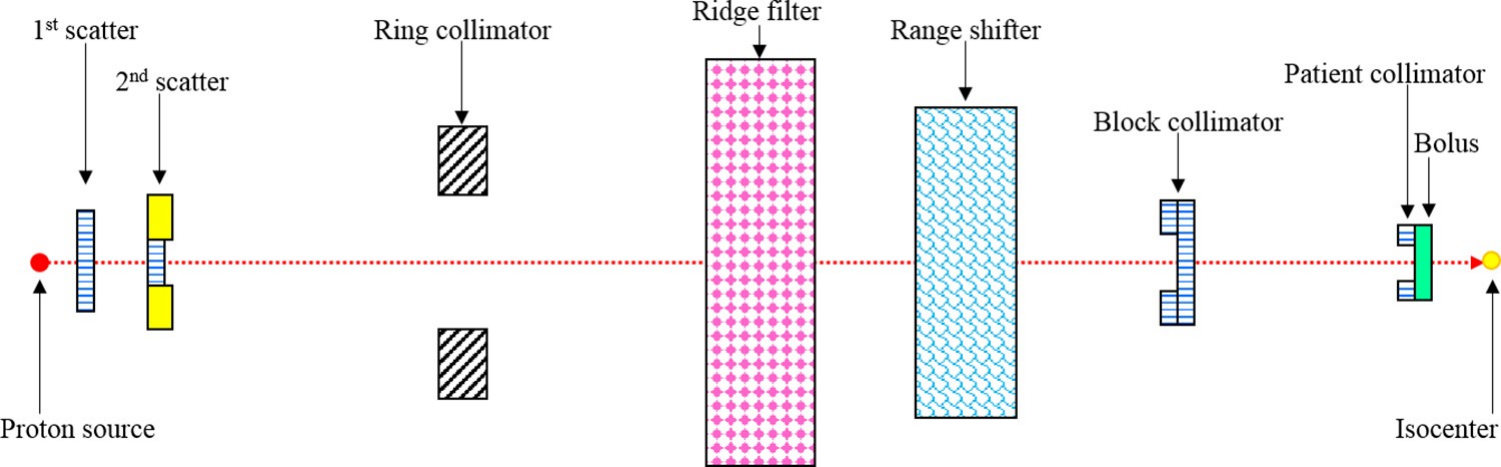
Schematic diagram showing modelled beamline port at NCCHE.

The same pediatric phantoms in Fig 1 were used in MC simulation of proton therapy. Port 2 of the NCCHE beamline was modelled using the MCNP code packages. In order to obtain the uniform lateral dose distribution, the double-scattering method was used and the modelled proton beamline was adopted from the previous work of Matsumoto *et al*. [28] that consisted of first and second scatter disks, 50 cm thick brass ring collimator, an aluminum ridge filter, a polymethyl methacrylate range shifter, block collimator, patient collimator and polyethylene bolus. The readers are referred to ref. [28] for the complete beamline setup at NCCHE. The modelled arrangement of the proton beamline is shown schematically in Fig 4. The track length estimate of energy tally was used to calculate the doses delivered to targeted and non-targeted organs. In the previous work of Sengbusch *et al*. [14], the 240 MeV proton source energy was found capable of treating 100% of patients in their study. Therefore, the energy of the proton source was set to be at 240 MeV.

### Dispersed dose conversion coefficient

The conversion coefficient was expressed as

$$F=\frac{D_{d}}{D_{t}}$$ (1)

where *F* was the dispersed dose conversion coefficient, *D*_*d*_ and *D*_*t*_ were the doses delivered to non-targeted tissues and the targeted tissue, respectively. The energy deposition in non-targeted and targeted tissues were obtained by using tally F6:P and F6:H for photons and protons, respectively. The doses in skeleton, skin, brain, spine, left lung, right lung, testes, colon and liver in phantoms of newborns, and 1, 5, 10 and 15-years olds were computed.

### Case study: Continuous hyper-fractionated accelerated radiotherapy treatment (CHART) of left lung

The present case study demonstrated the practical use of *F* values in pediatric radiation therapy. Here we chose a continuous hyper-fractionated accelerated radiotherapy treatment (CHART) schedule as an example which delivered 54 Gy to the targeted region within the left lung in 36 fractions, with 3 fractions per day over 12 days [35, 36]. Two assumptions were made, namely, (i) the same treatment plan was applicable for all pediatric phantoms and (ii) the same treatment plan was used in both photon and proton therapy. These assumptions facilitate comparisons among the doses delivered to non-targeted organs for different phantom ages and for two different radiation therapy treatment modes (i.e., photon and proton therapy). The doses dispersed to the brain, spine, left and right lung were calculated.

## Results and discussion

### Conversion coefficients in photon and proton radiation therapy

In all simulated cases of photon and proton irradiation of different pediatric phantoms, the same linear accelerator head and proton beamline were used. The most important components in the present study were the linear accelerator head and the proton beamline which were employed to produce a collimated photon and proton beam for therapeutic purposes, respectively. In our previous work, our Varian 2300 linear accelerator model [22] was benchmarked against previously reported results. The absorbed doses in targeted organs, i.e., testes (*P*_*1*_), colon (*P*_*2*_), liver (*P*_*3*_), left lung (*P*_*4*_) and brain (*P*_*5*_), for five different pediatric ORNL phantoms are shown in Tables 1 and 2 for photon and proton irradiation, respectively. These values correspond to the numerical values of *D*_*t*_ in Eq (1).

**Table 1:** Absorbed photon doses in targeted organs at different irradiation positions *P*_*1*_ to *P*_*5*_ obtained using a 6 MV linear accelerator beam.

Irradiation position	*P*_*1*_	*P*_*2*_	*P*_*3*_	*P*_*4*_	*P*_*5*_
Targeted tissue	Testes	Colon	Liver	Left lung	Brain
Age 15 absorbed dose (Gy/photon)	3.69×10^−16^	2.06×10^−16^	2.54×10^−16^	2.48×10^−16^	2.22×10^−16^
Age 10 absorbed dose (Gy/photon)	3.59×10^−16^	2.09×10^−16^	2.61×10^−16^	2.55×10^−16^	2.25×10^−16^
Age 5 absorbed dose (Gy/photon)	3.56×10^−16^	2.35×10^−16^	2.72×10^−16^	2.61×10^−16^	2.29×10^−16^
Age 1 absorbed dose (Gy/photon)	3.41×10^−16^	2.58×10^−16^	2.85×10^−16^	2.71×10^−16^	2.37×10^−16^
Newborn absorbed dose (Gy/photon)	3.23×10^−16^	2.31×10^−16^	2.82×10^−16^	2.78×10^−16^	2.56×10^−16^

**Table 2:** Absorbed proton doses in targeted organs at different irradiation positions *P*_*1*_ to *P*_*5*_ obtained using 240 MeV NCCHE beam.

Irradiation position	*P*_*1*_	*P*_*2*_	*P*_*3*_	*P*_*4*_	*P*_*5*_
Targeted tissue	Testes	Colon	Liver	Left lung	Brain
Age 15 absorbed dose (Gy/proton)	6.87×10^−13^	1.43×10^−13^	1.93×10^−13^	2.57×10^−13^	2.03×10^−13^
Age 10 absorbed dose (Gy/ proton)	6.84×10^−13^	1.80×10^−13^	2.89×10^−13^	2.74×10^−13^	2.15×10^−13^
Age 5 absorbed dose (Gy/ proton)	6.72×10^−13^	2.21×10^−13^	3.88×10^−13^	3.48×10^−13^	2.37×10^−13^
Age 1 absorbed dose (Gy/ proton)	6.66×10^−13^	2.51×10^−13^	5.91×10^−13^	5.73×10^−13^	3.39×10^−13^
Newborn absorbed dose (Gy/ proton)	6.53×10^−13^	3.13×10^−13^	5.92×10^−13^	6.47×10^−13^	7.32×10^−13^

The obtained dose values in the targeted organs (except at *P*_*1*_ for testes) showed that the absorbed photon and proton doses in general increased in targeted organs with a decrease in the patient age; this was mainly due to the reduced organ sizes and masses in younger patients. The absorbed dose in the testes in general decreased with a decrease in the patient’s age mainly due to variations in the geometry and size that significantly affected the efficiency of interactions between the radiation (either photon or proton) and the testes located outside the bodies of the male patients, which only allowed the photons or protons to either interact or penetrate with reduced scattering probability. Moreover, the relative statistical uncertainties associated with the absorbed photon doses in targeted organs were found to range from 0.81 to 0.19% for photon and proton irradiation. Interestingly, the absorbed proton doses were higher than photon doses, which was explained by their larger linear energy transfer (LET) when compared to that of photons.

**Fig 5:**
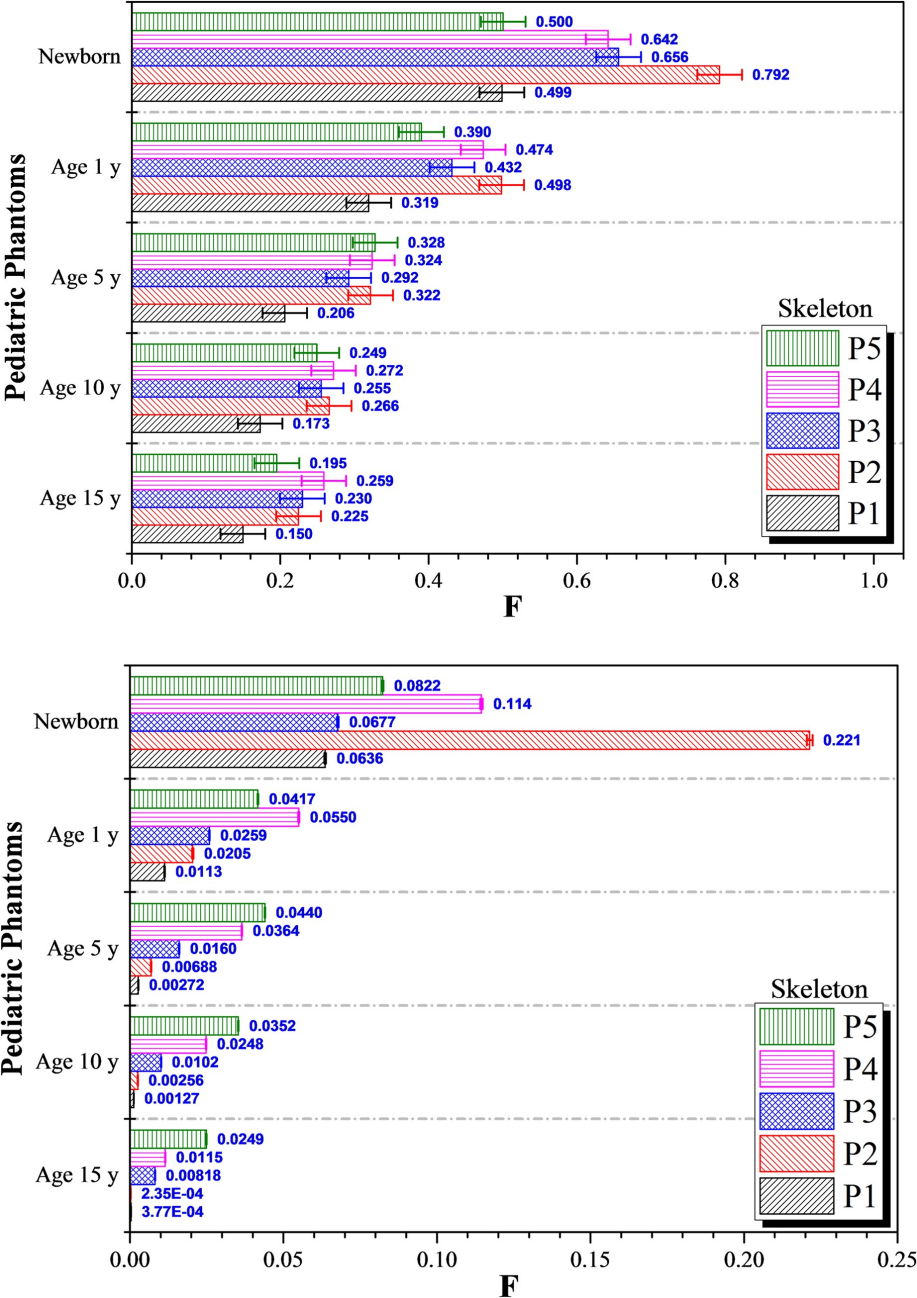
*F* values for skeleton at different irradiation locations *P*_*1*_ to *P*_*5*_ using (a) 6 MV linear accelerator beam and (b) proton beam. The error bars represented the associated statistical uncertainties obtained from MC simulations.

**Fig 6:**
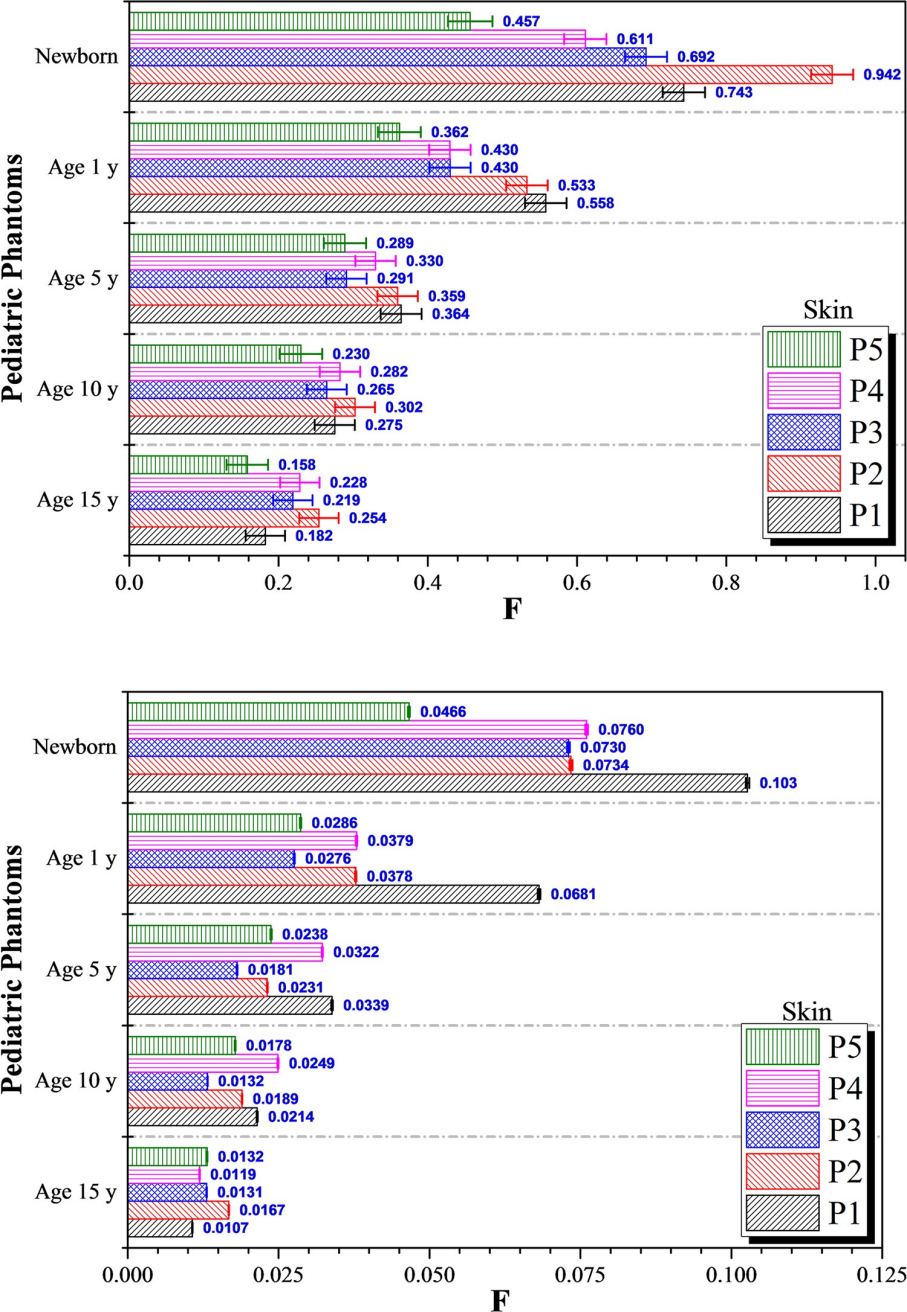
*F* values for skin at different irradiation locations *P*_*1*_ to *P*_*5*_ using (a) 6 MV linear accelerator beam and (b) proton beam. The error bars represented the associated statistical uncertainties obtained from MC simulations.

The variations in *F* for skeleton and skin at different irradiation positions for the five different pediatric phantoms are shown in Figs 5 and 6, respectively. In general, the *F* values obtained from proton irradiation (see Fig 5B) were significantly lower when compared to those obtained from photon irradiation (see Fig 5A). This showed that proton scattering within the patient was much lower compared to scattering of photons in the patient’s body; this so-called “limited scattering” was attributed to the large mass of protons, which made them tend to follow a somewhat straight-line trajectory. Fig 5A shows that variations in the absorbed photon doses in the skeleton are almost independent of the location of the targeted organ for phantoms of 15, 10 and 5-years old; this could be well observed from the ratio between the largest and smallest *F* values for the skeleton. However, this independency is no longer observable for proton irradiation (see Fig 5B); this could be observed from the variation between the maximum and minimum *F* values for skeleton for each pediatric phantom during proton irradiation. The photon beam delivered larger doses to the skeleton irrespective of the irradiation position, which was due to intense scattering. On the contrary, the proton beam had lower lateral scattering so the dose delivered to the skeleton would vary with the size of the pediatric patient. For younger patients, the small lateral scattering of protons would still deliver some dispersed dose to the skeleton. As such, the absorbed proton doses in the skeleton would depend on the location of the targeted organs for younger patients, which was in contrast to the case of photon beam showing independence of absorbed photon doses in the skeleton with the location of the targeted organ. However, as the overall body size of the patient increases with age, lateral scattering of protons would become less significant and thus the dose delivered to the skeleton would become negligible.

For the phantoms corresponding to 15, 10 and 5-years olds, considering different photon irradiation positions, the largest *F* values in the skeleton were 0.259, 0.272 and 0.332, respectively, while the smallest *F* values were 0.150, 0.173 and 0.292, respectively. In other words, the largest and smallest *F* values were not significantly different from one another, so the dispersed photon doses delivered to the skeleton were nearly independent of the irradiation position on the human phantom. On the other hand, again for the phantoms corresponding to 15, 10 and 5-years olds, considering different proton irradiation positions, the largest *F* values in the skeleton were 0.0249, 0.0352 and 0.044, respectively, while the smallest *F* values were 2.35×10^−4^, 0.00127 and 0.00272, respectively. Significant variations were thus observed among the *F* values for the skeleton when different proton-beam irradiation positions were chosen. Interestingly, for the 15, 10 and 5-years olds, the largest *F* values for skeleton were obtained for proton irradiation of the brain, where energy deposition in the skull by high-LET protons was inevitable. The main reason behind the disappearance of this independency was the “limited scattering” of protons when compared to photons, which made the dispersed doses location dependent, in contrast to the situation of photons which sparsely deposited energy through scattering within the patient’s body.

However, for 1-year olds and newborns, the photon doses dispersed to the skeleton differed noticeably with irradiation positions. The maximum *F* values for 1-year olds and newborns were 0.498 and 0.792, respectively, while the minimum values were 0.319 and 0.499, respectively. The *F* values for the skeleton were relatively higher for 1-year olds and newborns for both photon and proton irradiation, which showed that the doses dispersed to the patients’ skeleton increased with decreasing patient age. The largest *F* values for the skeleton occurred in newborns, being 0.792 for photon irradiation and 0.221 for proton irradiation, with delivered photon and proton doses to the human skeleton at 79.2% and 22.1% of the total absorbed photon and proton dose in colon (i.e., the targeted organ), respectively. The lowest *F* value corresponded to the desirable situation of lowest dispersed photon and proton doses. From Fig 5A, the lowest *F* value was ~0.5 (i.e., the most desirable case). Therefore, the lowest dispersed photon dose in skeleton in newborns was half the total dose delivered to the testes (*P*_*1*_) or the brain (*P*_*5*_). However, this value could be further reduced to 6.36% and 8.22% of the total dose delivered to the testes (*P*_*1*_) or the brain (*P*_*5*_) if proton irradiation was adopted.

The variations in *F* for the skin from different irradiation positions for the five considered ages using photon and proton beams are shown in Fig 6A and 6B, respectively. Generally speaking, the *F* values for proton irradiation were relatively much smaller than those for photon irradiation. The variations in the *F* values for the skin using photon irradiation followed a similar trend as those for skeleton (see Fig 2A). The *F* values were effectively independent of irradiation positions for phantoms corresponding to 15, 10 and 5-years olds. Similarly, the *F* values for the skin from proton irradiation were found to almost exhibit an independency of irradiation positions for age 15, 10 and 5 phantoms. This independency reflected the pervasive nature of the skin, which would be the target of protons during proton irradiation no matter where the proton beam was pointing, so the deposited proton dose in the skin would not vary significantly.

In contrast, the variations would increase for 1-year olds and newborns. The differences between the highest and lowest *F* values for the skin using photon irradiation for 15, 10 and 5-years olds were 0.096, 0.072 and 0.298, respectively. The corresponding differences for proton irradiation were 0.006, 0.012 and 0.016 for 15, 10 and 5-years olds, respectively. However, the differences for 1-year olds and newborns increased to 0.196 and 0.485, respectively, for photon irradiation and to 0.041 and 0.056, respectively, for proton irradiation. The relatively large *F* values for the skeleton and the skin for 1-year olds and newborns were due to their small overall body size, in such a way that the primary particles would interact with other non-targeted organs throughout the phantoms. It can be concluded that the doses dispersed to non-targeted organs in 1-year old and newborn patients would be significant when compared to older patients (i.e., 5-years old and beyond). Considering the relatively larger dispersed doses to non-targeted organs for 1-year old and newborn patients, there certainly would be undesirable clinical implications. In previous study by Paulino [37], several strategies to limit the side effects in children resulted from radiotherapy were reported. Some of these strategies might be applicable to treatments using protons as well, which included delaying the treatment until the child got older, decreasing the doses and volumes of radiotherapy and incorporating chemotherapy in the treatment (sequential chemoradiation), and changing the radiotherapy fractionation.

**Fig 7:**
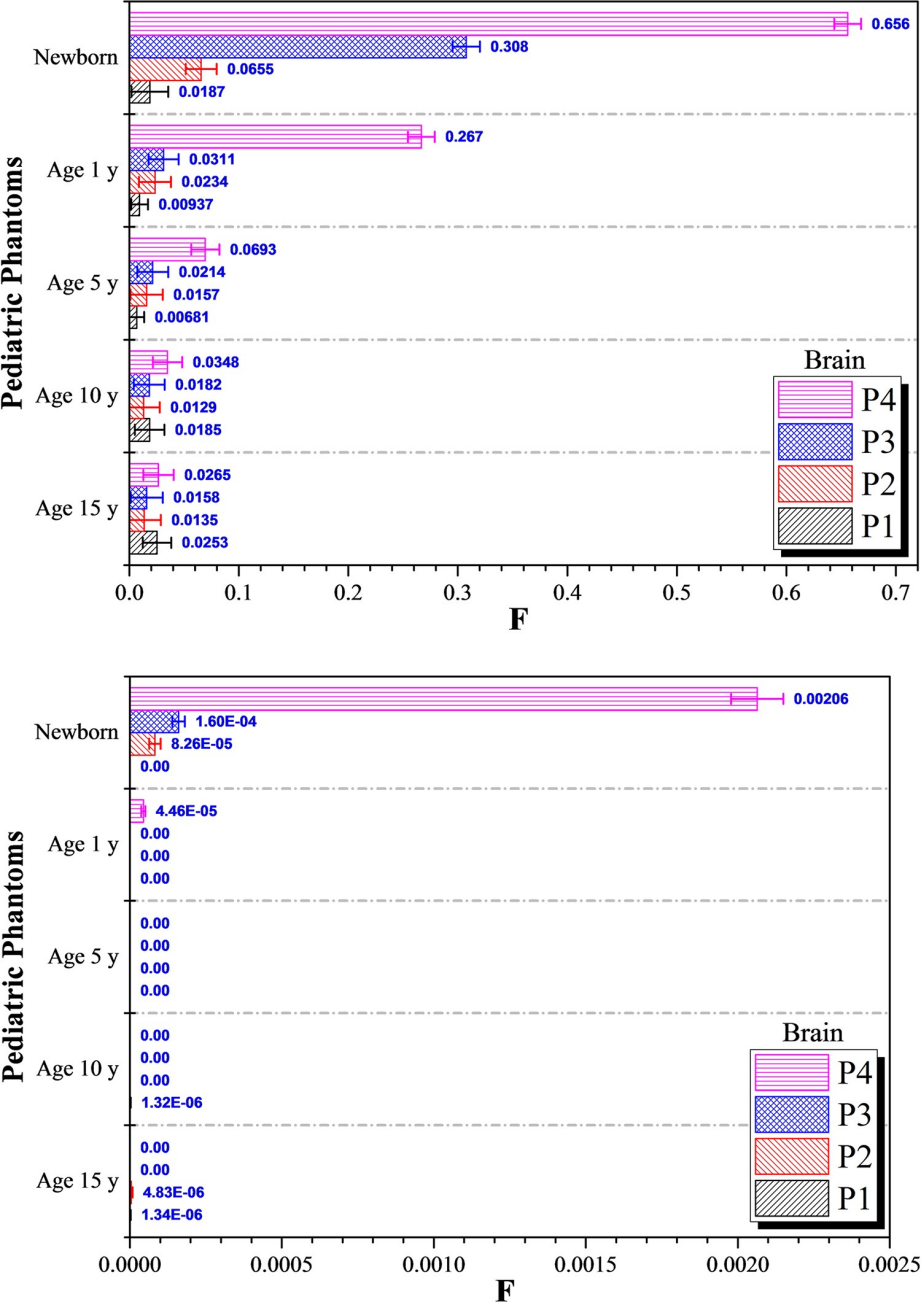
*F* values for the brain at different irradiation locations *P*_*1*_ to *P*_*5*_ using (a) 6 MV linear accelerator beam and (b) proton beam. The error bars represented the associated statistical uncertainties obtained from MC simulations.

The variations in *F* values for the brain for the five different pediatric phantoms are shown in Fig 7. The results for the irradiation position *P*_*5*_ (i.e., brain) are not shown since *F* = 1 at *P*_*5*_. The differences between the highest and lowest *F* values for 15, 10 and 5-years olds undergoing photon irradiation were 0.013, 0.022 and 0.058, respectively. These differences were significantly reduced when proton irradiation was employed (see Fig 7), which were 3.49×10^−6^, 1.32×10^−6^ and 0, respectively. Remarkably, proton irradiation resulted in negligible dispersed doses in the brain. In contrast, the highest photon dose dispersed to the brain was obtained during irradiation of the left lung in a newborn (i.e., irradiation position *P*_*4*_), which corresponded to 65.6% (cf. 0.206% for proton irradiation). The relatively lower photon and proton doses dispersed to the brain for 15, 10 and 5-years olds were mainly due to the larger phantom sizes when compared to 1-year olds and newborns. However, the dispersed doses would only be noticeable during irradiation of the left lung for 1-year olds and newborns. In contrast, for a newborn patient, irradiation of the liver and left lung led to noticeable photon and proton doses dispersed to the brain (see Fig 7A and 7B). The doses dispersed to the brain increased as the irradiation position got closer to the patient’s head and the increase was noticeable for 1-year old and newborn patients.

**Fig 8:**
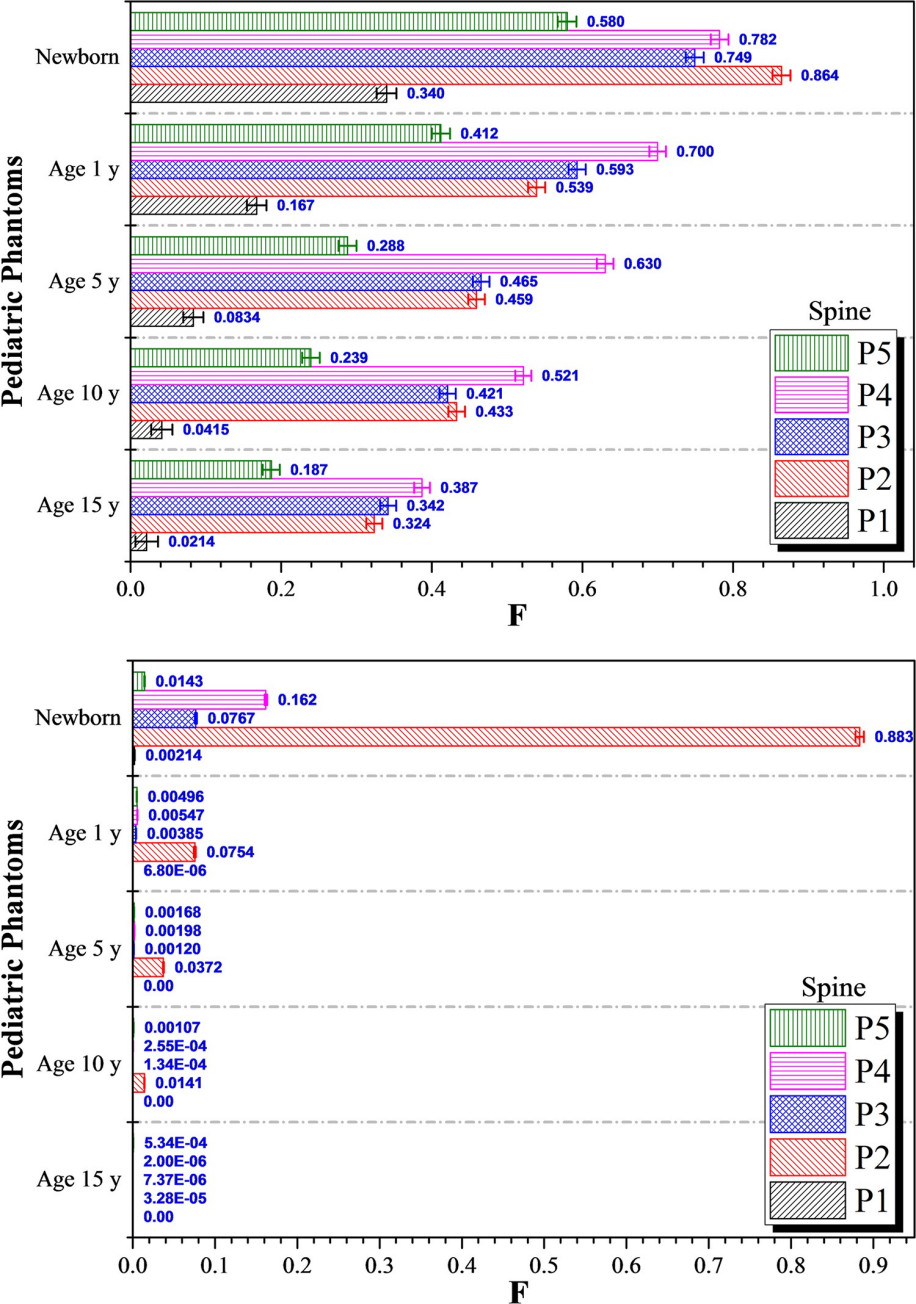
*F* values for the spine at different irradiation locations *P*_*1*_ to *P*_*5*_ using (a) 6 MV linear accelerator beam and (b) proton beam. The error bars represented the associated statistical uncertainties obtained from MC simulations.

The variations in *F* values for the spine for photon and proton irradiations are shown in Fig 8A and 8B, respectively, for 15, 10, 5 and 1-year olds, as well as newborns. As regards photon irradiation, variations in the dispersed photon dose in almost all phantoms were noticeable for all irradiation positions *P*_*1*_ to *P*_*5*_. In particular, the *F* values increased significantly for 1-year olds and newborns when compared to 15, 10 and 5-years olds. Interestingly, as regards proton irradiation, the dispersed proton doses in almost all phantoms were negligible, except those in newborns (see Fig 8B). Again, a smaller body size led to a larger dispersed dose. The contributions from scattered radiation to the dispersed doses in different organs were more prominent for 1-year olds and newborns, since the organs were located closer to one another when the overall body mass and size were smaller. For 15, 10, 5 and 1-year olds, the largest *F* values for the spine for photon irradiation were 0.387, 0.521, 0.630 and 0.700, respectively, obtained during irradiation of the left lung (*P*_*4*_ position). In contrast, the highest and second highest *F* values for the spine were 0.864 and 0.782 obtained during photon irradiation of the colon (*P*_*2*_) and left lung (*P*_*4*_), respectively, for newborns (see Fig 8A). The highest *F* values during proton irradiation of 15, 10, 5 and 1-year olds were 5.34×10^−4^, 0.0141, 0.0372 and 0.0754, respectively. As regards proton irradiation of newborn patients, the largest and second largest *F* values for the spine found were 0.883 and 0.162 which were obtained during proton irradiation of the colon (*P*_*2*_) and left lung (*P*_*4*_), respectively. The major portion of the spine was located along the central/middle axis of the human body, so the radiation doses dispersed from the irradiation points *P*_*2*_ (colon) and *P*_*4*_ (left lung) could reach the spine more effectively when compared to the farther irradiation positions *P*_*1*_ (testes) and *P*_*5*_ (brain). As expected, irradiation at *P*_*1*_ and *P*_*5*_ led to smaller *F* values.

**Fig 9:**
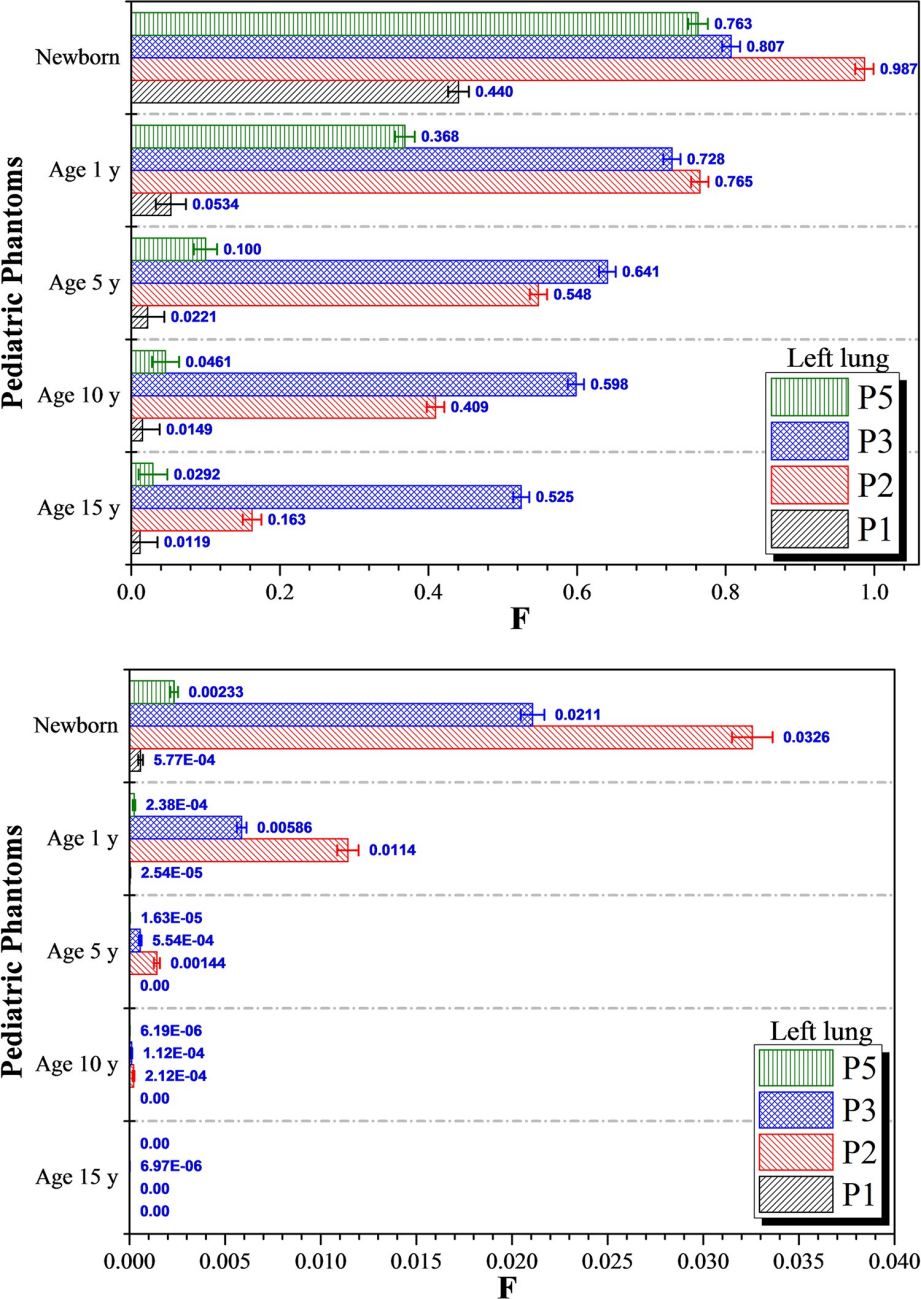
*F* values for the left lung at different irradiation locations *P*_*1*_ to *P*_*5*_ using (a) 6 MV linear accelerator beam and (b) proton beam. The error bars represented the associated statistical uncertainties obtained from MC simulations.

The variations in *F* values for the left lung for photon and proton irradiation are shown in Fig 9A and 9B, respectively. The *F* values for the left lung at irradiation position *P*_*4*_ are not shown as *F* = 1. Generally speaking, for both photon and proton irradiation, the highest *F* values for the left lung were obtained for irradiation positions *P*_*2*_ (colon) and *P*_*3*_ (liver). Dispersion of photons and generated electrons in the surrounding tissues were the main reasons behind the large *F* values for irradiation positions *P*_*2*_ and *P*_*3*_. The dispersed photon doses in the left lung were negligible for irradiation positions *P*_*1*_ and *P*_*5*_ when considering the results for 15, 10, 5 and 1-year olds (see Fig 9A). For proton irradiation, the *F* values for the left lung for 15, 10 and 5-years olds were negligible and almost no proton doses were dispersed to the left lung for the mentioned age groups. The proton dose dispersed to the left lung would be notable for 1-year olds and newborns (see Fig 9B). As regards photon irradiation, the lowest and highest *F* values for the left lung during irradiation of newborns were 0.440 and 0.987, respectively. Therefore, the photon dose dispersed to the left lung would be relatively significant regardless of irradiation positions for newborn patients. As regards proton irradiation, the lowest and highest *F* values during irradiation of newborn patients were 5.77×10^−4^ and 0.0326, respectively. This demonstrated that the choice of proton therapy could significantly reduce the dispersed doses to non-targeted organs, which was in contrast to conventional photon therapy, where particle dispersion was the main cause of dose dispersion to surrounding organs. This dispersion would be more significant for younger patients with smaller body size. For example, the incident photon with specific initial energy (*E*_*0*_) would undergo fewer interactions with surrounding tissues to reach the neighboring organs, so higher-energy photons and generated electrons could deposit larger amounts of energy to neighboring non-targeted organs.

**Fig 10:**
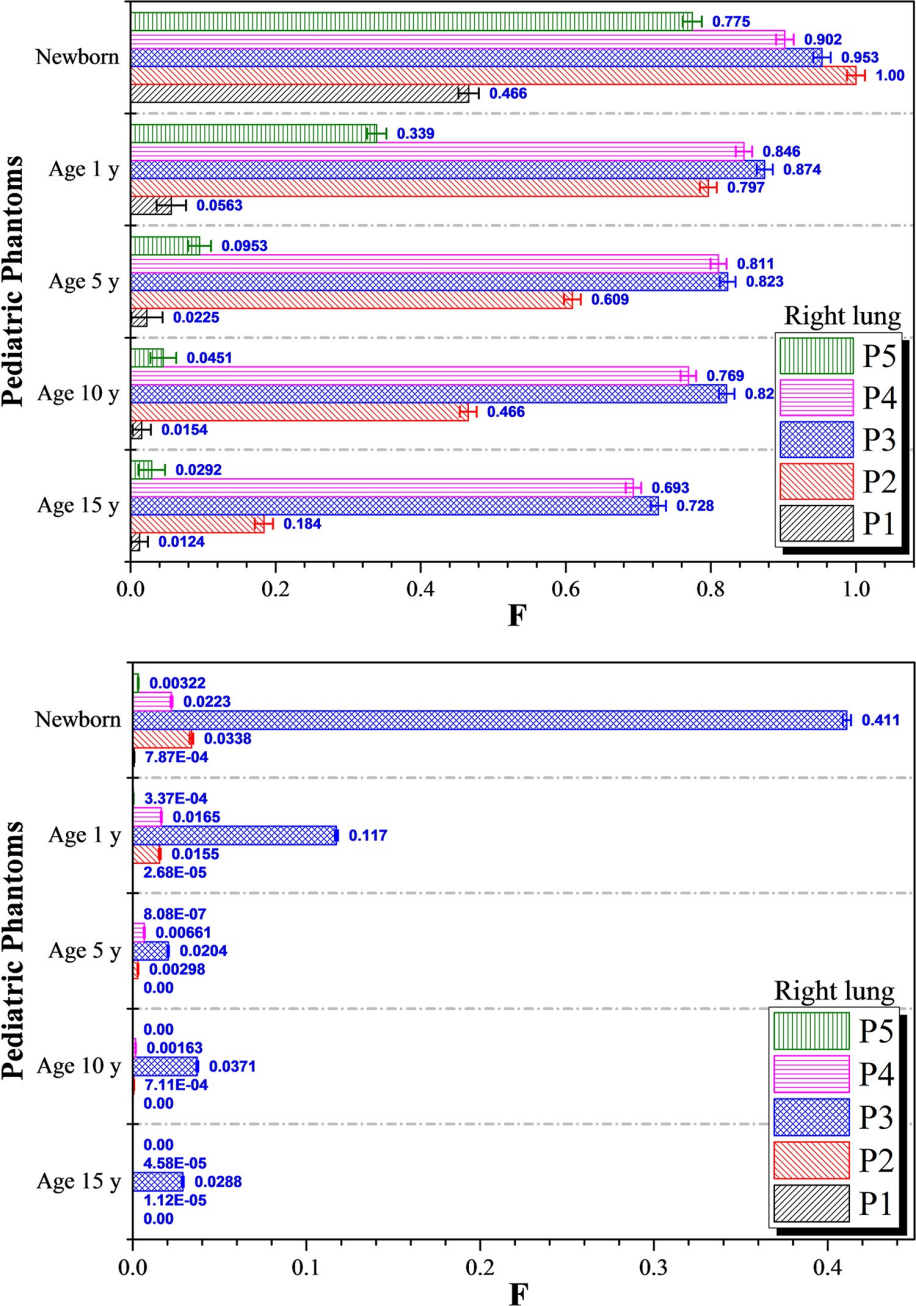
*F* values for the right lung at different irradiation locations *P*_*1*_ to *P*_*5*_ using (a) 6 MV linear accelerator beam and (b) proton beam. The error bars represented the associated statistical uncertainties obtained from MC simulations.

Lastly, variations in *F* values for the right lung for five irradiation positions for photon and proton irradiations are shown in Fig 10A and 10B, respectively. In general, the *F* values obtained for proton irradiation were lower compared to those for photon irradiation. Considering 15, 10, 5 and 1-year olds, the largest and second largest *F* values were obtained during irradiation of the liver (*P*_*3*_) and left lung (*P*_*4*_), respectively. The *F* values increased with decreasing patient age, showing that the dose dispersed to the right lung was more pronounced in younger patients. In our previous work [22] considering irradiation of an adult male, the largest and second largest *F* values were obtained for the right lung during irradiation of the left lung (*P*_*4*_) and liver (*P*_*3*_), which were different from the trend obtained here for pediatric phantoms. The difference was attributed to the reduced phantom height for younger patients, which was more significant than lateral changes. This made the axially dispersed particles more energetic as they underwent a smaller number of collisions to reach the right lung when the beam was initially pointing at the liver. The highest *F* values in the right lung were obtained in 1-year olds and newborns. The highest *F* values in 1-year olds were 0.874 and 0.117 for photon and proton irradiation, respectively, while the values in newborns were 1.00 and 0.411, respectively. This showed that the choice of proton irradiation could limit the dispersed doses for younger patients, but in newborns 41.1% of the dose delivered to the liver would be deposited into the right lung.

### The paired student’s t-test

There were a total of 28 *F* values for every age group both for photon and proton irradiations (shown in Figs 5–10), that were obtained using Eq (1). We performed paired t-tests on the corresponding 28 values for photons and protons for each age group, and concluded that the percentage of scattered doses from photon irradiations were much larger than those from proton irradiations with *p* values smaller than 9.14×10^−10^, 1.43×10^−7^, 6.51×10^−7^, 2.64×10^−6^ and 3.89×10^−12^, for 1, 5, 10, 15-year olds and newborns, respectively.

### Case study: Continuous hyper-fractionated accelerated radiotherapy treatment (CHART) of left lung

**Fig 11:**
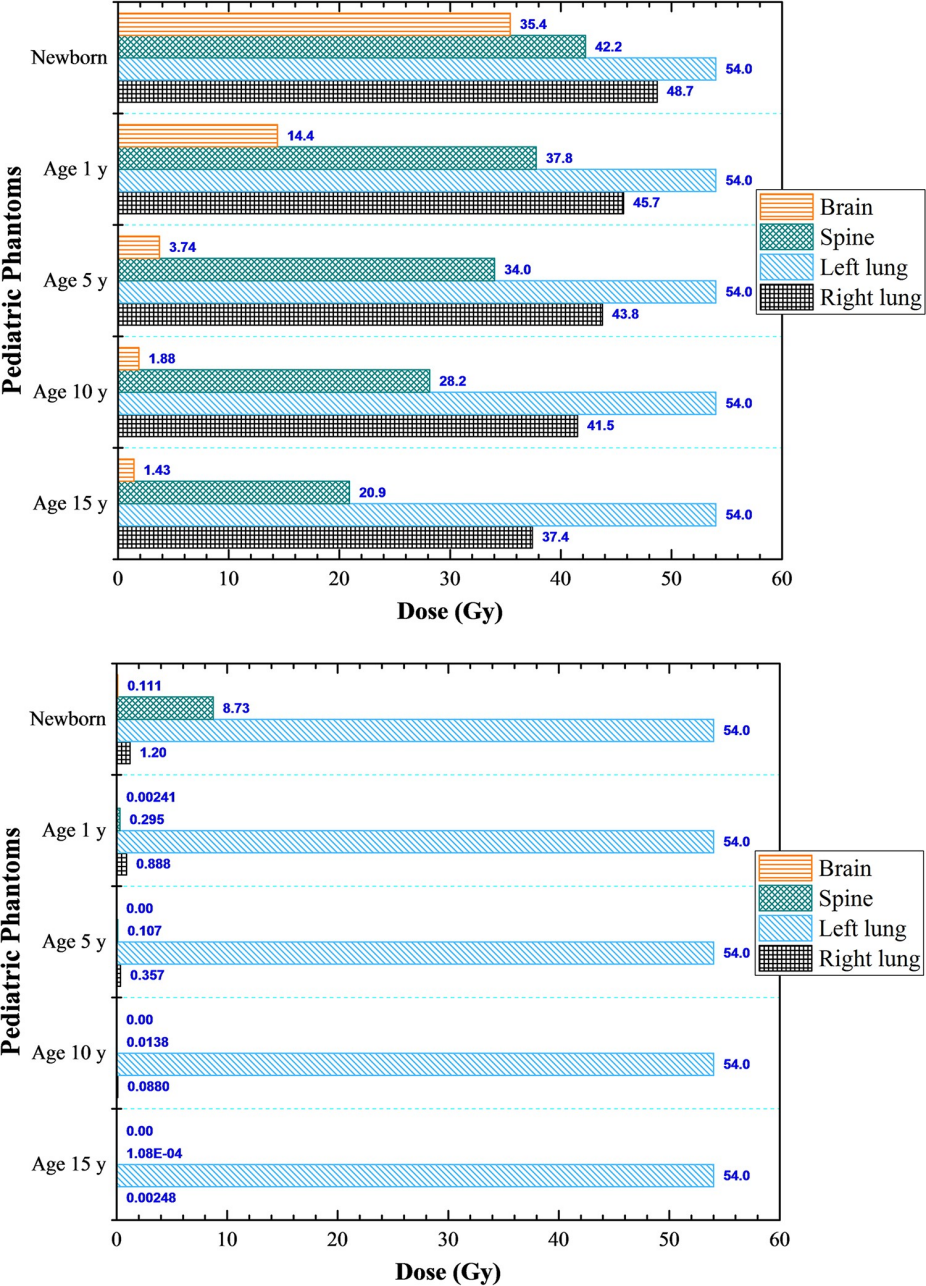
Dispersed doses delivered to non-targeted organs during the case study involving CHART (continuous hyper-fractionated accelerated radiotherapy) treatment of left-lung cancer using (a) 6 MV linear accelerator beam and (b) proton beam.

The photon and proton doses are shown in Fig 11A and 11B, respectively. The doses dispersed to non-targeted organs were significantly smaller for proton irradiation (see Fig 11B). In general, the doses delivered to non-targeted organs increased for younger patients. For example, the lowest dispersed photon doses during irradiation of the left lung were delivered to the brain, which were 1.43, 1.88, 3.74, 14.4 and 35.4 Gy for 15, 10, 5, 1-year olds and newborns, respectively. The photon doses delivered to the spine for 15, 10, 5, 1-year olds and newborns were 20.9, 28.2, 34.0, 37.8 and 42.2 Gy, respectively. For a comparison, the corresponding proton doses delivered to the spine were 1.08×10^−4^, 0.0138, 0.107, 0.295 and 8.73 Gy, respectively. The doses dispersed to non-targeted organs for photon irradiation were due to scattering of primary photons and secondary electrons from body tissues as well as the surroundings, which were limited in case of proton irradiation so the dispersed doses for proton therapy were much lower than those for conventional photon therapy.

### Dispersion of primary photons and secondary electrons

**Fig 12:**
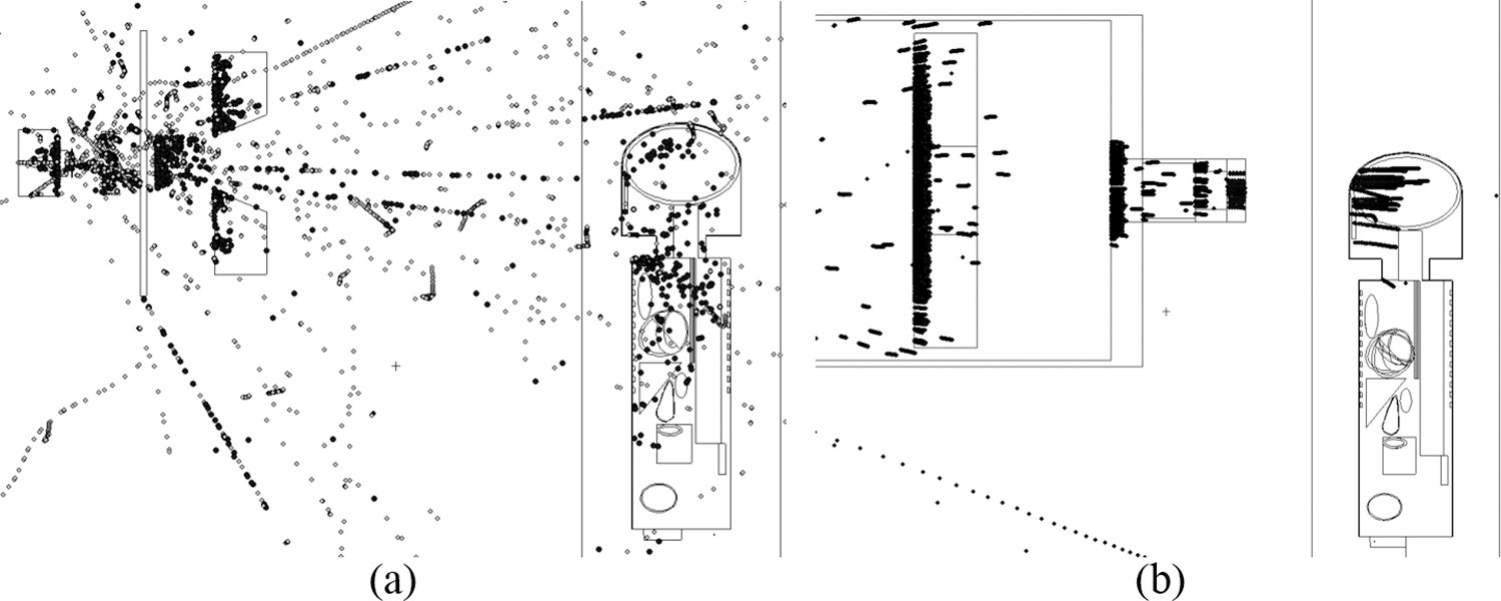
Dispersion of particles during irradiation of a 5-years old using (a) 6 MV linear accelerator beam and (b) proton beam.

Dispersion of photons and protons during irradiation of brain (*P*_*5*_) for a 5-years old are shown in Fig 12A and 12(B). The results highlighted the significance of severe particle dispersion during photon therapy in younger patients. In contrast, proton therapy did not result in such severe particle scattering. It is remarked that such snapshots could help understand the differences between photon and proton therapy modes in terms of particle transport and scattering.

## Conclusions

The present work showed the importance of conversion coefficients for dispersed dose (*F*) during pediatric radiotherapy using photon and proton irradiation. The *F* values for the skeleton, skin, brain, spine, left and right lungs were determined for five different irradiation positions on ORNL phantoms of 15, 10, 5 and 1-year olds as well as newborns using photon and proton beams. The *F* values for almost all non-targeted organs increased with decreasing age and the *F* values were significantly higher for 1-year old and newborn patients due to their smaller body size. Moreover, the *F* values were significantly lower during proton therapy of pediatric patients, which was attributed to the large mass of protons that led to lesser particle scattering within the pediatric patients. The lowest photon doses dispersed to the skeleton of newborns were half the total doses delivered to the testes (*P*_*1*_) or the brain (*P*_*5*_). For proton irradiation of 15, 10 and 5-years olds, the highest *F* values for skeleton were 0.0249, 0.0352 and 0.044, respectively. For a comparison, the lowest *F* values for the skin were 2.35×10^−4^, 0.00127 and 0.00272, respectively. The *F* values for the skin in the case of photon irradiation were almost independent of irradiation positions on 15, 10 and 5-years olds. In relation, variations in the *F* values for the skin for proton irradiation were found to almost exhibit an independency for 15, 10 and 5-years olds. Dispersed photon doses (i.e., *F* values) for the brain were found noticeable during irradiation of the left lung for a 1-year-old and newborn, with *F* values of 26.7% and 65.6%, respectively, of the total dose delivered to the left lung. In contrast, the proton doses dispersed to the brain were negligible for proton irradiation. The highest *F* value for the brain during proton therapy of a newborn reached the maximum of 0.206%, which demonstrated that proton therapy of pediatric cancer patients could significantly limit the dispersed dose. The highest *F* value during photon irradiation of the spine was 86.4% of the dose delivered to the colon (*P*_*2*_) for a newborn. For proton irradiation of a newborn, the doses dispersed to the spine were 88.3% and 16.2% of the proton dose delivered to the colon (*P*_*2*_) and left lung (*P*_*4*_), respectively.

The NRUrad input codes for different ORNL pediatric phantoms developed in the present work provided a useful tool to determine case-specific conversion coefficients for both photon and proton therapy. The feasibility of simulating realistic treatment conditions using parallel (MPI) computing on multi-core computer cluster was also verified.
